# Anti-inflammatory effect of Resolvin D1 on LPS-treated MG-63 cells

**DOI:** 10.3892/etm.2019.8021

**Published:** 2019-09-18

**Authors:** Dan Cao, Jing Pi, Yihong Shan, Yuping Tang, Ping Zhou

Exp Ther Med 16: 4283-4288, 2018; DOI: 10.3892/etm.2018.6721

Following the publication of this paper, the authors have realized that the name of their institute, as presented in the affiliations, should have been changed from “Children's Hospital Affiliated to Nanjing Medical University” to “Children's Hospital of Nanjing Medical University”. Therefore, the authors' affiliations, and the affliation address, should have appeared as follows:

DAN CAO*, JING PI*, YIHONG SHAN*, YUPING TANG and PING ZHOU

Department of Orthopedics, **Children's Hospital of Nanjing Medical University**, Nanjing, Jiangsu 210009, P.R. China

*Contributed equally

The authors confirm that there are no further errors in the study, and all the authors agree to this correction. The authors regret this error in the affiliations, and apologize for any inconvenience caused.

